# GrapHi-C: graph-based visualization of Hi-C datasets

**DOI:** 10.1186/s13104-018-3507-2

**Published:** 2018-06-29

**Authors:** Kimberly MacKay, Anthony Kusalik, Christopher H. Eskiw

**Affiliations:** 1 Department of Computer Science, University of Saskatchewan, 110 Science Place, Saskatoon, SK S7N 5C9 Canada; 2 Department of Food and Bioproduct Science, University of Saskatchewan, 51 Campus Drive, Saskatoon, SK S7N 5A8 Canada

**Keywords:** Hi-C, Whole-genome contact maps, Data visualization, Graphs

## Abstract

**Objectives:**

Hi-C is a proximity-based ligation reaction used to detect regions of the genome that are close in 3D space (or “interacting”). Typically, results from Hi-C experiments (contact maps) are visualized as heatmaps or Circos plots. While informative, these visualizations do not directly represent genomic structure and folding, making the interpretation of the underlying 3D genomic organization obscured. Our objective was to generate a graph-based contact map representation that leads to a more intuitive structural visualization.

**Results:**

Normalized contact maps were converted into undirected graphs where each vertex represented a genomic region and each edge represented a detected (intra- and inter-chromosomal) or known (linear) interaction between two regions. Each edge was weighted by the inverse of the linear distance (Hi-C experimental resolution) or the interaction frequency from the contact map. Graphs were generated based on this representation scheme for contact maps from existing fission yeast datasets. Originally, these datasets were used to (1) identify specific principles influencing fission yeast genome organization and (2) uncover changes in fission yeast genome organization during the cell cycle. When compared to the equivalent heatmaps and/or Circos plots, the graph-based visualizations more intuitively depicted the changes in genome organization described in the original studies.

**Electronic supplementary material:**

The online version of this article (10.1186/s13104-018-3507-2) contains supplementary material, which is available to authorized users.

## Introduction

One of the major problems in the genomic era is understanding how genomes are organized and chromosomes are folded within cells. Genomic organization, specifically the close physical proximity of genetic elements located either distally on the same chromosome or located on different chromosomes, greatly impacts cellular processes such as transcription, replication and recombination [1]. The close physical proximity of two genetic elements is often referred to as an “interaction”. Knowledge of what interactions are occurring and how they are mediated is essential to understanding genome functions such as gene expression regulation. The biological assay Hi-C [2, 3] (or one of its derivatives [4–7]) can be used to detect interactions between regions of the genome on the same chromosome (intra-chromosomal or *cis*-interactions) or different chromosomes (inter-chromosomal or *trans*-interactions).

Briefly, Hi-C involves chemically cross-linking regions of the genome that are in close spatial proximity. Restriction enzyme digestion and ligation is then preformed on the cross-linked regions to generate chromatin/DNA complexes which can be identified by high-throughput sequencing. The resultant sequence reads are mapped to a reference genome [8] to determine the frequency with which each interaction occurs within the population of cells. The results of a Hi-C experiment are often encoded as a symmetric $N \times N$ matrix (contact map) where *N* is the number of genomic “bins” into which the genome is partitioned. Each genomic bin represents a linear region of genomic DNA, where the number of bins is approximately equal to the total genome size divided by the experimental resolution. For instance, a Hi-C experiment in fission yeast that is able to attain 10 kB resolution will generate 1258 genomic bins, each representing roughly 10 kB of linear DNA sequence. Each cell ($CM_{i,j}$) of the contact map records the interaction frequency between genomic bins *i* and *j*. Inherent systematic biases within the whole-genome contact map are dampened by normalizing the interaction frequencies. Typically, an ICE [9] or Knight-Ruiz [10, 11] normalization is applied to the raw data resulting in fractional interaction frequencies.

In a typical workflow, normalized contact maps are initially visualized as heatmaps or Circos [12] plots before further downstream analysis and 3D modelling [13]. While informative, these visualizations do not intuitively represent the complex organization and folding of the genome in 3D space. This makes it difficult to quickly understand the underlying 3D genome organization represented by the contact map. Our hypothesis is that representing and visualizing contact maps as a graph will lead to a more intuitive structural visualization of Hi-C data when compared to typical methods. We have developed a protocol called GrapHi-C (pronounced “graphic”) for visualizing Hi-C data as a graph. GrapHi-C utilizes a graph-based representation of a contact map and existing interactive tools for a more intuitive structural visualization of Hi-C data. We applied GrapHi-C to two existing datasets to demonstrate the improvements it can bring to interpreting Hi-C data.

## Main text

### Results and discussion

#### Graph-based representation

**Fig. 1:**
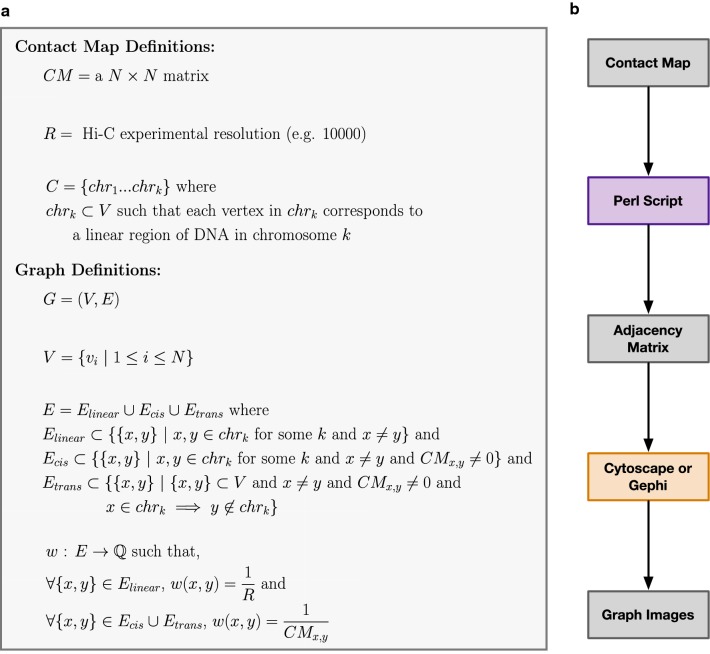
A formal description of the graph representation and workflow used by GrapHi-C. **a** The mathematical model used to represent a contact map as an undirected graph in the GrapHi-C protocol. **b** Overview of the GrapHi-C protocol. Each step of the workflow is indicated in a box where the different colours correspond to: data input and output (grey), developed Perl script (purple), and an existing tool (orange). An option for scaling the interaction frequencies is available in the developed Perl script if future studies wish to use it

In GrapHi-C visualizations, a contact map is translated into an undirected graph where each genomic bin is represented as a vertex and the detected or known interactions between bins are represented as undirected weighted edges. Specifically, edges represent linear, *cis*- and *trans*-interactions. Each edge is weighted with the inverse of the experimental resolution (for linear interactions) or interaction frequency (for *cis*- and *trans*-interactions). The edges representing *bonafide* in vivo linear connections between bins (i.e. the linear extent of the chromosome) add additional biological constraints. A formal description of the graphical representation used in GrapHi-C is presented in Fig. 1a.

#### Visualization protocol

A Perl script was developed that is able to convert a normalized contact map into an adjacency matrix based on the graph representation described above (available at: https://github.com/kimmackay/GrapHi-C, or Additional file 1). The output of this script can then be input into a tool like Cytoscape [14] or Gephi [15] to generate a structural visualization. Utilizing existing network visualization tools is advantageous since there are multiple plug-ins and layouts available which allow for flexibility in visualization and subsequent analysis.

It should be noted that Hi-C data cannot be directly input into tools like Cytoscape or Gephi. CytoHiC is the only existing Cytoscape plug-in for Hi-C data. It is used for pairwise comparisons of contact maps based on genetic landmarks such as methylation [16] and would provide a complementary analysis to GrapHi-C. The current version of CytoHiC is not compatible with the latest major release of Cytoscape (released February 2013) and the plug-in does not appear to be actively maintained. Unlike GrapHi-C, CytoHiC does not include edges representing linear interactions. It also utilizes a different edge weight equation to incorporate genetic landmarks into its comparison.

#### Applications

To demonstrate the value of the GrapHi-C visualization protocol, it was utilized to visualize contact maps from existing fission yeast datasets where (1) fission yeast mutants were studied to determine principles of genomic organization [17] and (2) synchronized fission yeast cells were used to track genomic organization throughout the cell cycle [18]. In each case, normalized fission yeast contact maps (10 kB resolution) were downloaded from the Gene Expression Omnibus database. The specific accession numbers are listed in “Availability of data and materials” section. These contact maps were transformed into adjacency matrices using the Perl script described above. The matrices were then input to the developed GrapHi-C protocol depicted in Fig. 1b. For comparison, the normalized fission yeast contact maps were also visualized as heatmaps and Circos plots. The heatmaps were generated using Java Treeview [19] which is the recommended visualization tool for contact maps generated from Hi-C data analysis pipelines [20]. The Circos plots were visualized in Cytoscape [14].

##### Application 1

**Fig. 2:**
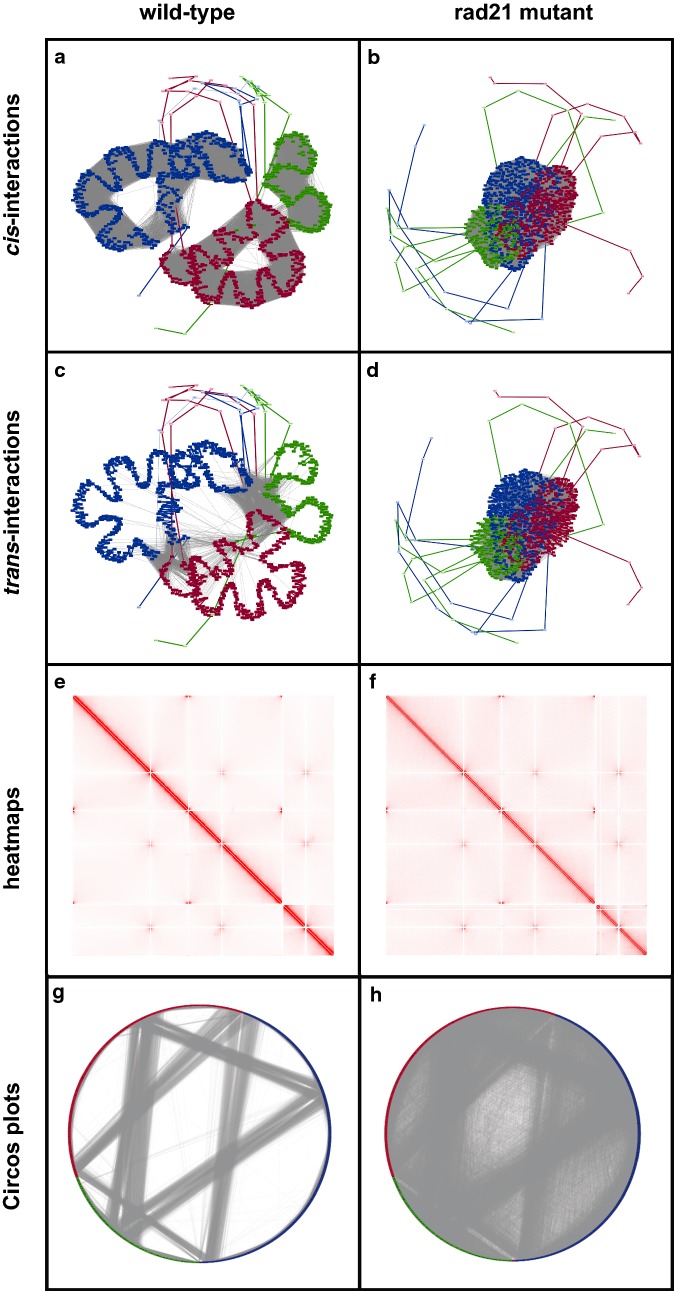
Comparison of GrapHi-C Visualizations, Heatmaps and Circos plots. Visualizations of the contact maps for the fission yeast *999a* wild-type and *rad21* mutant are displayed in the left and right columns, respectively. ** a**–**d** The GrapHi-C visualizations where vertices and linear interactions were coloured according to their corresponding chromosome (chromosome 1: blue, chromosome 2: red, chromosome 3: green). The grey dashed lines represent *cis*-interactions (**a**, **b**) and *trans*-interactions (**c**, **d**). Graphs were visualized in Cytoscape using a edge-weighted spring embedded layout. **e**, **f** The heatmaps generated with Java Treeview that correspond to the contact maps. The opacity of a cell is directly related to the frequency of the interaction. **g**, **h** The Circos plots that correspond to the contact maps. Circos plots were visualized in Cytoscape. Vertices were coloured according to their corresponding chromosome (chromosome 1: blue, chromosome 2: red, chromosome 3: green) and grey lines represent an interaction between two vertices

To determine if GrapHi-C can recapitulate the differences between wild-type and mutant fission yeast genome organization identified by Mizuguchi et al. [17], the *999a* wild-type and the *rad21* mutant adjacency matrices were visualized in Cytoscape using an edge-weighted spring embedded layout with the default parameters (Fig. 2a–d). Vertices along the periphery of the graph images correspond to genomic bins that represent centromere and telomere regions. Since these regions are highly condensed and repetitive (making the DNA difficult to assay and map), no interaction data was reported for them. All edges (corresponding to *cis*-, *trans*- and linear interactions) were used to generate the GrapHi-C images. To create the images in Fig. 2, nodes were manually coloured according to their corresponding chromosome and edges were hidden or revealed to highlight the *cis*- and *trans*-interactions.

For comparison, heatmaps (Fig. 2e, f) and Circos plots (Fig. 2g, h) for the *999a* wild-type and the *rad21* mutant contact maps were generated. These images represent the standard, existing approach for the visualization of Hi-C data. They clearly demonstrate how the traditional forms of visualization do not intuitively represent the complex organization and folding of the genome in 3D space (Fig. 2e–h). This makes it challenging to generate hypotheses about how differences in the wild-type and mutant contact maps are reflected in genome organization. On the contrary, the GrapHi-C visualizations (Fig. 2a–d) clearly highlight the loss of structural globules (intra-chromosomal structures) and the greater intermingling of chromosomes in the mutant strain that was described in the original study [17]. The resultant visualizations for the *rad21* mutant strain (Fig. 2b, d) appear to be very similar since the mutant contact map has smaller interaction frequency values (as compared to the wild type) due to a greater intermingling of chromosomes. This results in the nodes being placed closer together in the edge-weighted spring embedded layout. The *rad21* interaction frequency values are not scaled in order to maintain consistency when comparing the wild-type and mutant strain visualizations. Overall, the GrapHi-C visualizations made it easier to quickly identify the principles of genome organization and associated biological effects of the *rad21* yeast mutant that were discovered in the original study. This suggests the developed graph-based representation and structural visualization is a valid way to represent contact maps.

##### Application 2

**Fig. 3:**
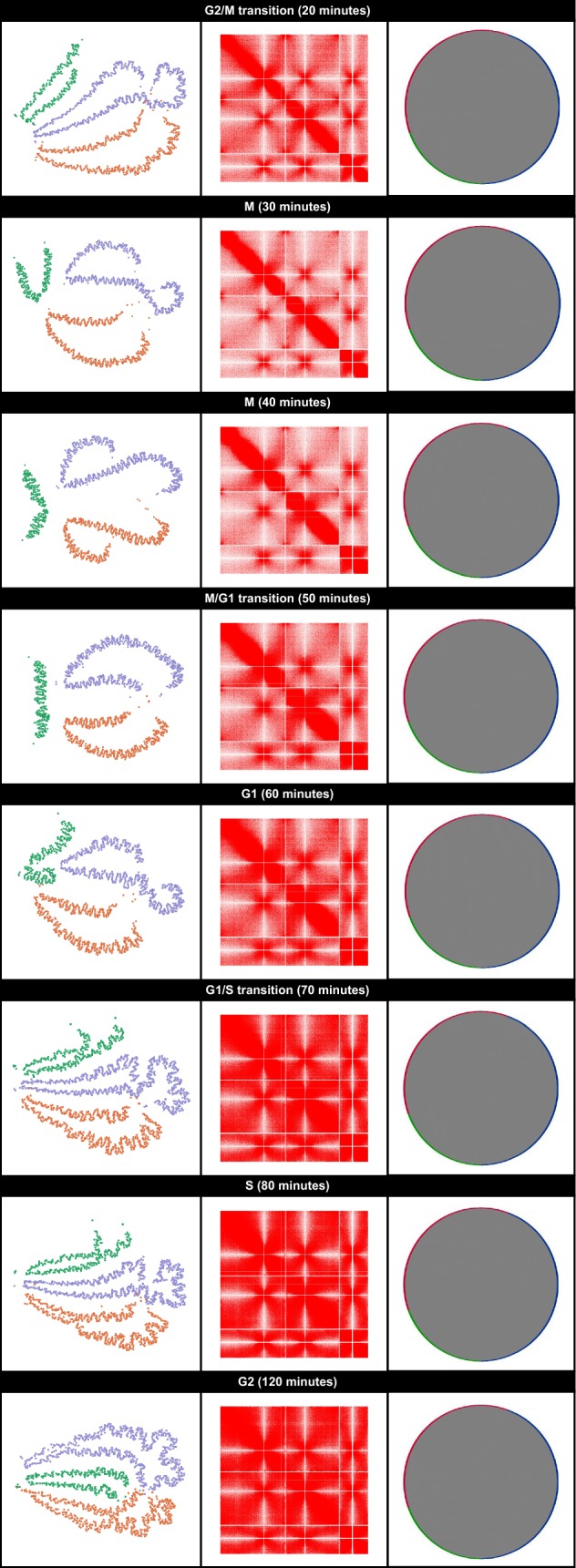
GrapHi-C visualizations for fission yeast contact maps at various stages of the cell cycle. Cell cycle labels and the corresponding time points are given on the top of each panel. GrapH-C images are presented in column 1. In these images, vertices were coloured according to their corresponding chromosome (chromosome 1: purple, chromosome 2: orange, chromosome 3: green) and edges were hidden. Graphs were visualized in Gephi using the ForceAtlas2 layout. The corresponding heatmaps generated by Java Treeview are in column 2. In these heatmaps, the opacity of a cell is directly related to the frequency of the interaction. Column 3 contains the Circos plots that correspond to the contact maps. Circos plots were visualized in Cytoscape. Vertices were coloured according to their corresponding chromosome (chromosome 1: blue, chromosome 2: red, chromosome 3: green) and grey lines represent an interaction between two vertices. Note that the area inside the circle appears to be solid grey due to the number (and subsequent density) of interactions in these datasets

To determine if the GrapHi-C protocol was able to identify the same cell cycle dependent alterations in genome organization described by Tanizawa et al. [18]. GrapHi-C images, based on the normalized contact maps for each time point, were visualized in Gephi using the ForceAtlas2 layout [21] (Fig. 3—column 1). Similarly to Application 1, all edges (corresponding to *cis*-, *trans*- and linear interactions) were used to generate the images. Nodes were manually coloured according to their corresponding chromosome and edges were hidden in the exported images for simplicity. GrapHi-C images containing all the edges for the 40, 60, 80 and 120 minute time points are provided in Additional files 2, 3, 4 and 5. For comparison, the heatmaps (Fig. 3—column 2) and Circos plots (Fig. 3—column 3) were generated based on the normalized contact maps for each cell cycle time point. As mentioned previously, these images represent the standard, existing approach for Hi-C data visualization.

In the original study, Tanizawa et al. [18] established that throughout the cell cycle, small domains approximately 50 kB in size are consistently present. During mitosis (M) the DNA condenses resulting in more *cis*-chromosomal interactions and fewer *trans*-chromosomal interactions. Not only are the GrapHi-C visualizations able to recapitulate these identified cell cycle dependant genomic alterations, they also intuitively highlight established features of fission yeast genomic organization. For instance, during fission yeast interphase (comprised of the G1, S and G2 phases) the chromosomes are organized in a polarized arrangement (*Rab1*-like configuration) where the centromeres of all three chromosomes are clustered at one end of the nucleus and the telomeres of chromosomes 1 and 2 cluster at the opposite end near the nuclear periphery [22]. Additionally, microscopy techniques have established that all three chromosomes are organized into distinct chromosome territories within the nucleus at all phases of the cell cycle [22–24]. These hallmarks of fission yeast genome organization are distinctly recapitulated in the GrapHi-C visualizations. Furthermore, the GrapHi-C visualizations clearly represent the established condensation of chromosomal DNA during mitosis and de-condensation during interphase [22]. Overall, GrapHi-C provides a more intuitive representation of how the chromosomes are organized within the nucleus during different phases of the cell cycle. The resultant images are informative additions which support the in-depth analysis performed in the original study.

### Conclusion

In this manuscript, we provide a protocol called GrapHi-C (pronounced “graphic”) for visualizing Hi-C data as a graph and developed a mathematical model for graph-based representations of contact maps. In addition to edges that represent the detected *cis*- and *trans*-interactions, we chose to include edges between each sequential genomic bin within a chromosome to better represent the linear extent of the genome. We developed a Perl script that can be used to convert a contact map into an adjacency matrix related to the developed graph-based representation. This matrix can then be input into a tool like Cytoscape or Gephi for structural visualization. Even though the graph-based representation seems straightforward, it is still novel in the genome structure community.

Overall, the developed GrapHi-C visualizations of the contact maps (compared to the equivalent heatmaps and Circos plots) made it easier to quickly identify the changes in genome organization identified in previous studies. Future work will focus on extending this visualization to allow for the vertices to be coloured according to complementary -omics datasets (such as gene expression, epigenetic markers or transcription factor binding sites) and produce a 3D graph-based visualization. Additionally, we will apply it to organisms with larger genomes to determine how well it scales to larger contact maps. Not only does the graph-based representation of Hi-C data lead to a more intuitive visualization, it also has the potential to lead to new ways of analyzing contact maps by leveraging tools and results from graph theory.

## Limitations

GrapHi-C has only been tested on Hi-C datasets from haploid organisms—it should also be applied to organisms with higher ploidies to establish the robustness of the workflow. Additionally, GrapHi-C needs to be tested on an unfavourable Hi-C dataset that contains a multitude of disparate proximity relationships. Finally, the effect of visualizing a Hi-C dataset with technical problems needs to be established.

## Availability of data and materials

The datasets supporting the conclusions of this article are available in the Gene Expression Omnibus database, [accession number: GSE56849; https://www.ncbi.nlm.nih.gov/geo/query/acc.cgi?acc=GSE56849, GSE93198; https://www.ncbi.nlm.nih.gov/geo/query/acc.cgi?acc=GSE93198]. The specific sample numbers for each application follow.

- Application 1
  - *999a* (GSM1379427)
  - *rad21* (GSM1379430)
- Application 2
  - 20 min (GSM2446256)
  - 30 min (GSM2446257)
  - 40 min (GSM2446258)
  - 50 min (GSM2446259)
  - 60 min (GSM2446260)
  - 70 min (GSM2446261)
  - 80 min (GSM2446262)
  - 120 min (GSM2446263)

*Software information*

Project Name: GrapHi-C (pronounced \graphic")

Project home page: https://github.com/kimmackay/GrapHi-C/

Archived version: v1.0.0

Operating system(s): Platform independent

Programming language: Perl

Other requirements: Not Applicable

License: This work is licensed under the Creative Commons Attribution-NonCommercial-ShareAlike 3.0 Unported License. To view a copy of this license, visit http://creativecommons.org/licenses/by-nc-sa/3.0/ or send a letter to Creative Commons, PO Box 1866, Mountain View, CA 94042, USA.

## Additional files

**Additional file 1.** Perl script used for converting a contact map into an adjacency matrix based on the graphrepresentation in Fig. 1a.

**Additional file 2.** GrapHi-C Visualization for Fission Yeast Contact Map During M Phase (40 min)In this image, vertices were coloured according to their corresponding chromosome (chromosome 1: purple,chromosome 2: orange, chromosome 3: green). The cis- and trans-interactions edges are depicted with grey lines.Due to the number (and subsequent density) of these lines, these appear to be a solid grey area. The graph wasvisualized in Gephi using the ForceAtlas2 layout.

**Additional file 3.** GrapHi-C Visualization for Fission Yeast Contact Map During G1 (60 min)In this image, vertices were coloured according to their corresponding chromosome (chromosome 1: purple,chromosome 2: orange, chromosome 3: green). The cis- and trans-interactions edges are depicted with grey lines. Due to the number (and subsequent density) of these lines, these appear to be a solid grey area. The graph wasvisualized in Gephi using the ForceAtlas2 layout.

**Additional file 4.** GrapHi-C Visualization for Fission Yeast Contact Map During S Phase (80 min)In this image, vertices were coloured according to their corresponding chromosome (chromosome 1: purple,chromosome 2: orange, chromosome 3: green). The cis- and trans-interactions edges are depicted with grey lines.Due to the number (and subsequent density) of these lines, these appear to be a solid grey area. The graph wasvisualized in Gephi using the ForceAtlas2 layout.

**Additional file 5.** GrapHi-C Visualization for Fission Yeast Contact Map During G2 (120 min)In this image, vertices were coloured according to their corresponding chromosome (chromosome 1: purple,chromosome 2: orange, chromosome 3: green). The cis- and trans-interactions edges are depicted with grey lines.Due to the number (and subsequent density) of these lines, these appear to be a solid grey area. The graph wasvisualized in Gephi using the ForceAtlas2 layout.

## Abbreviation

3D: three-dimensional
