# Supplementary figures and images for: GrapHi-C: graph-based visualization of Hi-C datasets
Source: BMC Res Notes. 2018 Jun 29;11:418. doi: 10.1186/s13104-018-3507-2 (PMC6025839; doi:10.1186/s13104-018-3507-2)

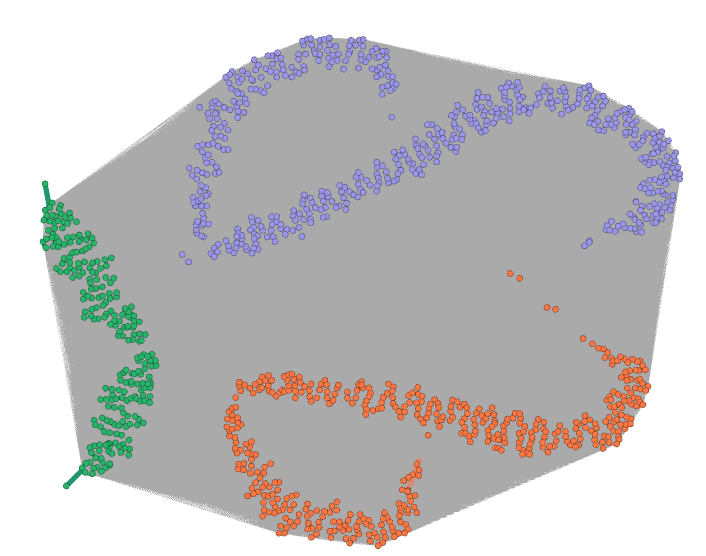

Supplement: Supplementary file 2 — Additional file 2. GrapHi-C Visualization for Fission Yeast Contact Map During M Phase (40 min)In this image, vertices were coloured according to their corresponding chromosome (chromosome 1: purple,chromosome 2: orange, chromosome 3: green). The cis- and trans-interactions edges are depicted with grey lines.Due to the number (and subsequent density) of these lines, these appear to be a solid grey area. The graph wasvisualized in Gephi using the ForceAtlas2 layout. [file 13104_2018_3507_MOESM2_ESM.png]

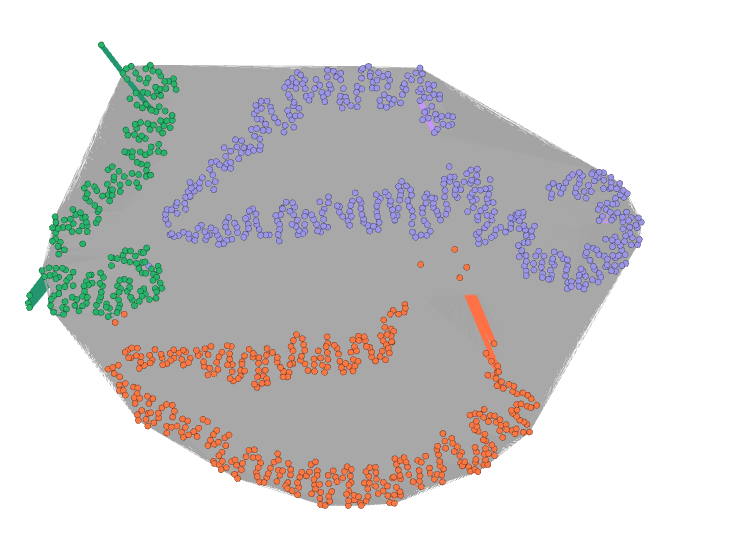

Supplement: Supplementary file 3 — Additional file 3. GrapHi-C Visualization for Fission Yeast Contact Map During G1 (60 min)In this image, vertices were coloured according to their corresponding chromosome (chromosome 1: purple,chromosome 2: orange, chromosome 3: green). The cis- and trans-interactions edges are depicted with grey lines. Due to the number (and subsequent density) of these lines, these appear to be a solid grey area. The graph wasvisualized in Gephi using the ForceAtlas2 layout. [file 13104_2018_3507_MOESM3_ESM.png]

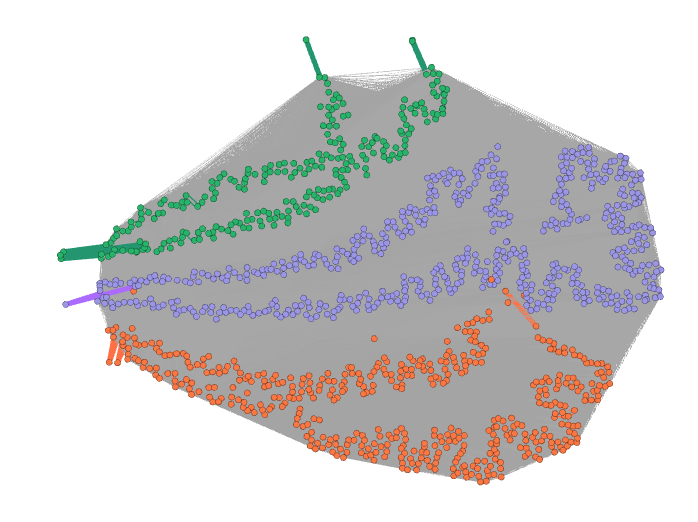

Supplement: Supplementary file 4 — Additional file 4. GrapHi-C Visualization for Fission Yeast Contact Map During S Phase (80 min)In this image, vertices were coloured according to their corresponding chromosome (chromosome 1: purple,chromosome 2: orange, chromosome 3: green). The cis- and trans-interactions edges are depicted with grey lines.Due to the number (and subsequent density) of these lines, these appear to be a solid grey area. The graph wasvisualized in Gephi using the ForceAtlas2 layout. [file 13104_2018_3507_MOESM4_ESM.png]

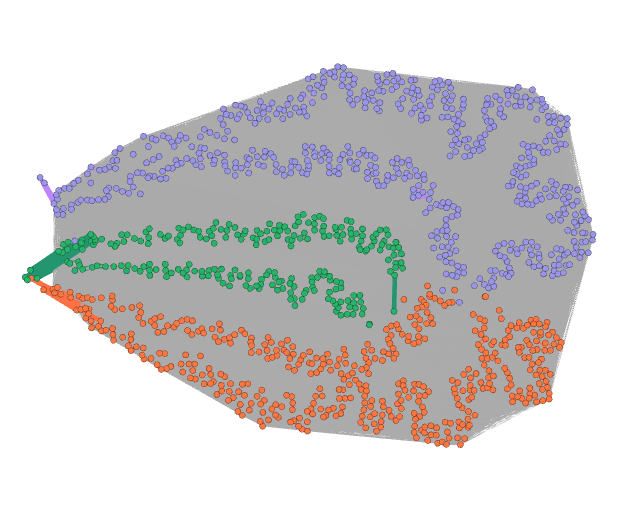

Supplement: Supplementary file 5 — Additional file 5. GrapHi-C Visualization for Fission Yeast Contact Map During G2 (120 min)In this image, vertices were coloured according to their corresponding chromosome (chromosome 1: purple,chromosome 2: orange, chromosome 3: green). The cis- and trans-interactions edges are depicted with grey lines.Due to the number (and subsequent density) of these lines, these appear to be a solid grey area. The graph wasvisualized in Gephi using the ForceAtlas2 layout. [file 13104_2018_3507_MOESM5_ESM.png]
